# E‐learning or lectures to increase knowledge about congenital heart disease in infants: A comparative interventional study

**DOI:** 10.1002/nop2.317

**Published:** 2019-07-01

**Authors:** Elin Hjorth‐Johansen, Dag Hofoss, Nina Margrethe Kynø

**Affiliations:** ^1^ Division of Paediatric and Adolescent Medicine Department of Neonatal Intensive Care Oslo University Hospital Oslo Norway; ^2^ Lovisenberg diaconal University College Oslo Norway

**Keywords:** congenital heart disease, education, e‐learning, haemodynamics, neonatal intensive care

## Abstract

**Aim:**

This project aimed to create, implement and evaluate an e‐learning course on nursing infants with congenital heart disease (CHD) and to measure its efficacy compared with classroom learning.

**Design:**

This is a comparative interventional study with two groups.

**Methods:**

The study involved 15 postgraduate students and 13 newly employed nurses. The learning outcome was computed as the difference between pre‐test and post‐test knowledge scores and analysed using *t* tests and multiple regression.

**Results:**

Both learning groups scored significantly higher 1 week after training. The improvement did not differ significantly between the groups when controlling for the years of experience in CHD nursing and the baseline knowledge score. Participants with higher baseline knowledge scores improved their scores less. Neither learning method was proven more effective than the other. Participants reported experiencing traditional classroom teaching as more positive, but e‐learning was more time effective.

## 1 INTRODUCTION

In Norway each year, about 750 infants are born with congenital heart disease (CHD) and around 100 infants with severe CHD undergo heart surgery in the neonatal period (Jortveit et al., 2016). All neonates with severe CHD are transferred to the Oslo University Hospital (OUH) for medical assessment and treatment immediately after birth.

High‐quality nursing of newborn infants with CHD demands complex knowledge and advanced nursing skills (Fleiner, 2006). Neonatal intensive care unit (NICU) nursing staff have a high level of turnover and new colleagues who often have little clinical experience in nursing infants with CHD (Aiken, Clarke, Sloane, Lake, & Cheney, 2008; Kerfoot, 2000; Khowaja‐Punjwani, Smardo, Hendricks, & Lantos, 2017). This leads to a continuous need to train staff on the different aspects of CHD, such as anatomy, haemodynamics, medical treatments, clinical nursing skills, the specific types of heart failure and signs and symptoms of infants with CHD.

## 2 BACKGROUND

Based on the need for high expertise and updated knowledge in the field of neonatal nursing, continuous education is among the highest priorities in the NICUs in Norway (Norwegian Directorate of Health, 2017). Computer‐based teaching programmes give the opportunity to new innovative methods that allow delivering of continuous education to both postgraduate students and new employed nurses. E‐learning is a rapidly growing teaching method in health care and has expanded the opportunities for flexible, convenient and interactive education (Lahti, Hätönen, & Välimäki, 2014). Reported benefits of e‐learning include flexibility, accessibility, satisfaction and cost‐effectiveness (Lahti et al., 2014).

Results from studies comparing the efficacy of e‐learning and traditional learning vary. Challenges in comparing learning effects from research reports include the heterogeneity in subjects, complexity of content and conceptual differences in e‐learning programs (Cook, Garside, Levinson, Dupras, & Montori, 2010; George et al., 2014; Lahti et al., 2014). The heterogeneity in subjects is reflected in a wide scope of nursing activities such as pain management (Keefe & Wharrad, 2012), basic life support (Moule, Albarran, Bessant, Brownfield, & Pollock, 2008), knowledge in anatomy and physiology (Kaveevivitchai et al., 2009), knowledge and performance of hand hygiene (Bloomfield, Roberts, & While, 2010), assessment and pressure ulcer classification (Bredesen, Bjøro, Gunningberg, & Hofoss, 2016) and knowledge of clinical nephrology (Segal et al., 2013). Knowledge has been measured by a wide range of methods like multiple‐choice questions, short essay questions, open‐ended or Likert‐type questions (George et al., 2014). Skills and satisfaction have also been tested by different approaches. Furthermore, the participants have had different time available to go through the courses, which lead to inequivalent exposure time to the interventions (Cook et al., 2010; George et al., 2014). However, several meta‐analyses suggest that e‐learning and traditional learning are equally efficient (George et al., 2014; Lahti et al., 2014; McCutcheon, Lohan, Traynor, & Martin, 2015).

E‐learning has been proposed as an efficient method to increase knowledge on nursing infants (Rouse, 2000). The subject of CHD is complex and difficult for students and new nurses to comprehend. The purpose of this project was to create, implement and evaluate an e‐learning course on haemodynamic understanding and nursing infants with CHD and to measure its efficacy compared with traditional face‐to‐face learning. The outcome measure was the increase in the knowledge score on a multiple‐choice test. The project also looked at how much study time the two learning groups used and whether the participants were more comfortable with e‐learning than with traditional learning.

Our research questions are the following:

- Which of the two learning methods:
  o increase the knowledge score most?
  o is the most time effective?
  o do the participants prefer?

## 3 THE STUDY

The aim of this project was to create, implement and evaluate an e‐learning course on nursing infants with CHD and to measure its efficacy compared with classroom learning.

### 3.1 Design

This is a comparative intervention study with two groups.

### 3.2 Methods

All students enrolled at the postgraduate course in neonatal nursing (a 60 credit points further education) at the Oslo Diaconal University College (LDUC), and all newly employed nurses (last 6 months) at the two neonatal departments at the OUH were invited to undergo a 1‐day CHD course. Volunteers were randomly assigned to e‐learning or classroom lecturing. The classroom lecture groups contained 10 students and five newly employed nurses, the e‐learning group six students and seven newly employed nurses.

Data were collected immediately before and after the course and 1 week after the course.

#### 3.2.1 Intervention

The intervention was developed in a collaborative project between LDUC and the children's department of OUH. A multiprofessional group of CHD expert physicians and nurses was established to ensure that the course covered all major aspects of CHD deemed relevant to nursing CHD infants in the OUH neonatal hospital departments. The course content consisted of core themes in haemodynamics and CHD neonatal nursing as defined by the expert group.

The group closely collaborated with an illustrator who made pictures and films to present the content pedagogically. Course development was inspired by Nokelainen's suggestions for pedagogical usability (Nokelainen, 2006). These include stating clear goals, breaking down the material into units, learner control by flexibility and interactivity with immediate response. The e‐learning course was a computer‐based package of five courses (Table 1), and the classroom learning group covered the same topics in six lectures of 45 min each. The e‐learning and classroom courses had the same content. Pictures and films in the PowerPoint slides were the same, but with text adapted either to the classroom lectures or the e‐learning course. The e‐learning course contained interactive questions which had to be completed successfully before the participants were allowed to proceed. The traditional learning included dialogue and lecturing and did not have the optional interactive questions.

**Table 1:** Name, content and learning goals of the five courses/lectures

Name of course/lecture	Learning goals
Normal circulation and electricity of the heart	To gain a basic understanding of heart anatomy and physiology
Transition from foetal circulation to normal circulation	To understand foetal circulation and the transition to normal circulation as a basis for understanding the haemodynamics of congenital heart defects
Haemodynamics in congenital heart disease (CHD)	To understand the concept of haemodynamics and pathophysiology and how structural abnormalities affect infants' circulation
Nursing infants with different kinds of heart failure	To understand heart failure development, causes and drug therapy; to become familiar with core nursing observation tasks and corresponding follow‐up actions; and to learn how to inform parents regarding their infants' condition
Observation and action when diagnosing CHD in the emergency room	To learn how to support investigation and stabilization of the circulation disorder by clinical observation, how to use monitoring equipment, how to secure intravenous inputs and how to administer vital drugs

The main differences between the course formats were that online students were able to proceed at their own pace, while classroom participants went at the same pace as the lecturer and that classroom participants, but not e‐learning participants, could discuss the course content with the teacher and the other students.

#### 3.2.2 Participants

In total, 26 postgraduate students in the LDUC neonatal nursing programme and 14 nurses employed during the last 6 months at the two sections at OUH NICU were invited to participate in the study. Out of these 40 registered nurses, 28 (70%) agreed to participate (15 students and 13 nurses).

#### 3.2.3 Setting

The intervention was conducted from 9–10 March 2016. Both groups received information on the nature of the study before answering the pre‐knowledge test. Participants signed informed consent forms, and the study was approved by the hospital's privacy protection supervisor. The e‐learning group worked on their course at their own pace in a computer laboratory at OUH. The other group received traditional lectures from an experienced nurse specialist in a classroom at OUH.

#### 3.2.4 The knowledge test

To our knowledge, no test targeting CHD nursing topics were available. The multiprofessional expert group developed a 36‐question multiple‐choice test containing central topics in the courses. Six nurses with different length of CHD‐experiences pilot tested the questionnaire to identify misunderstandings and errors and a few minor changes were made. Thirty questions had one correct answer out of three or four options (Vyas & Supe, 2008). Six questions had multiple correct answers, and respondents were made aware of this in the questionnaire.

To reduce the number of guesses and accidental high scores, we included an “I don't know” option among the response categories. Participants were instructed to tick this category instead of guessing if they did not know the answer (van Mameren & van der Vleuten, 1999).

The knowledge test that was used as measure instrument was not validated but contained central knowledge questions that nurses working with this population should have. This assessment was made by the expert group.

**Figure 1:**
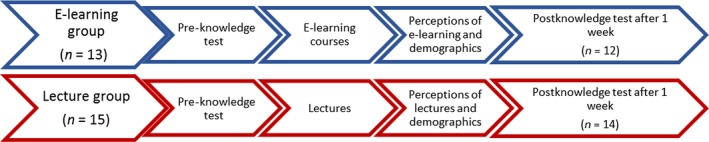
Completion of the study

Participants completed the pre‐test immediately prior to the CHD course. After the intervention at the end of the course, they also completed a questionnaire about demographics and their perception of the course. The participants received sealed envelopes containing the 1 week postcourse knowledge test (identical to the immediate postcourse test) and were told to open the envelopes and answer the test questions 1 week after the course (Figure 1). A telephone text message was sent to remind the participants to do the post‐test. Participants returned their answers either by posted mail or by depositing them (in sealed envelope) in a box at the nurse educators' office. Response rates at post‐test were 14 in the traditional learning group and 12 in the e‐learning group.

#### 3.2.5 Data collection and variables

Our primary outcome measure was the knowledge score in the multiple‐choice test. The secondary outcome measures were (a) the participants' satisfaction with the learning method and (b) the time spent on learning.

Pre‐ and post‐training knowledge scores were computed as the number of correct answers to the 36 multiple‐choice questions before and 1 week after the course. For questions that had several correct answers, all correct ticks were counted. The maximum score was 50 points. Demographic data and data from the pre‐test were collected on the course day, whereas post‐test data were collected between 16–18 March 2016.

The learning outcome was computed as the difference between pre‐ and post‐test knowledge scores. Participants' satisfaction with the instructional method (e‐learning or traditional learning) and familiarity with the course content were measured on a 5‐point scale ranging from *not at all* (0 point) to *a very high degree (4 point)*.

#### 3.2.6 Statistical analyses

Learning group differences in background characteristics such as the baseline knowledge score, number of years of experience with CHD neonates and satisfaction with the learning method were tested using independent samples *t* tests. The difference in learning outcomes between the e‐learning group and the traditional learning group, as well as the demographic differences between the two groups, was tested using one‐way analysis of variance (ANOVA).

The relationship of the learning outcome with the instructional method was studied using multiple linear regression. The relatively low number of cases in this study (*N* = 28) narrowed the scope of control variables to be included in the learning outcome explanatory model. A rule of thumb is that the number of explanatory variables should not exceed one tenth of the number of observations (Katz, 2006). The regression model linking the learning outcome to the teaching method therefore included only the two control variables that we considered most likely to affect the learning outcome: the participants' baseline knowledge of and clinical experience with nursing CHD infants. The participants' baseline knowledge of nursing CHD infants can affect the learning outcome because those who had more knowledge about CHD in infants before the course started might not have benefited from the courses as much as the less well trained. On the other hand, previous clinical experience with nursing CHD infants gives opportunities to place the theoretical content from the courses in context.

All calculations were done with the Statistical Package for the Social Sciences version 24. Differences and relationships with *p* values not exceeding 0.05 were deemed significant.

## 4 RESULTS

Compared with the traditional learning group, the e‐learning group had more experience working with infants with CHD, but there were no differences between the groups in pre‐ or post‐test knowledge scores. The traditional learning group was more satisfied with the learning method than the e‐learning group, but the participants in the e‐learning group completed the courses more quickly (Table 2).

**Table 2:** Demographics, perception of learning method and test scores

	Classroom learning group (*N* = 15)	E‐learning group (*N* = 13)	*p*
Years of experience with infants with congenital heart disease	0.1 Range: 0–1.5 *SD*: 0.39 CI_95_: 0–0.3	0.9 Range: 0–3.5 *SD*: 1.35 CI_95_: 0.1–1.7	0.045
Pre‐test score	22.9 Range: 5–41 *SD*: 10.5 CI_95_: 17.0–28.7	27.8 Range: 14–41 *SD*: 10.5 CI_95_: 21.5–34.2	0.220
Post‐test score	36.5 (*N* = 14) Range: 21–45 *SD*: 6.9 CI_95_: 32.6–40.3	38.0 (*N* = 12) Range: 25–49 *SD*: 8.2 CI_95_: 32.8–43.2	0.600
To which degree did you find the used learning method positive?^a^	3.5 (*N* = 13) Range: 3–4 *SD*: 0.5 CI_95_: 3.2–3.9	2.8 Range: 1–4 *SD*: 1.0 CI_95_: 2.2–3.4	0.023
How much time was used for the course?	4.5 hr (270 min) (Length of course: 6 courses × 45 min)	2.1 hr (122 min) Range: 76–240 *SD*: 49.1 min CI_95_: 98–157	<0.001

a Scale: 0–4: Not at all (0), To a small degree (1), To some degree (2), To a high degree (3), To a very high degree (4).

The score of the face‐to‐face learning group increased from 22.9–36.5 (*p* < 0.001), while that of the e‐learning group increased from 27.8–38.0 (*p* < 0.001). Both groups significantly increased their scores from pre‐ to post‐test.

The improvement was more pronounced in the traditional classroom instruction group (Mean improvement = 13.60, *SD*: 8.86) than in the e‐learning group (Mean improvement = 9.58, *SD*: 6.01), but the difference was not significant.

The *F* test of the explanatory model provided significant evidence that this group of selected explanatory variables was related to CHD knowledge score improvement (*p*_F_ < 0.001) and the model's *r* ^2^ score showed that the regression model explained 51% of the variance in score improvement.

The baseline knowledge score was significantly related to the participants' knowledge score improvement. Those who scored one point higher at *t*_1_ showed, as expected, less improvement: 0.588 points lower (*p* < 0.001) on average. Course participants' number of years of clinical CHD experience was not significantly related to the improvement in their knowledge test scores (Table 3).

**Table 3:** Multiple linear regression relationships of learning outcome with teaching format, baseline knowledge score and length of congenital heart disease (CHD) clinical experience

Variables	*β*	*p*
Teaching method (1 = e‐learning, 0 = classroom)	−2.260	0.340
Baseline knowledge score (5–41)	−0.588	<0.001
Years of CHD clinical experience (0–3.5)	1.797	0.170
Constant	26.860	<0.001

Note

Model goodness‐of‐fit statistics: *F* = 10.181 (*df* = 3), *p*_F_ < 0.001; *r* ^2^ = 0.570, $r_{\text{adj}}^{2}$ = 0.514.

The difference in test score improvement between the two learning groups was not significant regardless of whether the baseline test score and clinical CHD experience were controlled for.

## 5 DISCUSSION

In this study, both e‐learning and classroom lectures produced significantly better knowledge scores, but the improvement did not differ significantly by the learning method. These findings are in line with some earlier research showing that e‐learning and lectures produce the same learning outcome in nurses (Lahti et al., 2014) and students in healthcare professions (George et al., 2014). As pointed out in the background section, these reviews are based on single studies that are heterogeneous in topics, methods and outcome measures (Cook et al., 2010). Therefore, our results, like those of other studies, may reflect the pedagogical quality of a particular e‐learning program or lecturer and thereby be difficult to generalize. Yet, such single study‐results add to the body of evidence regarding e‐learning. In CHD especially, our results support the findings of an evaluation of a former CHD course, which concluded that e‐learning and traditional learning strategies are comparable. In addition, they also found that adding e‐learning after traditional learning leads to significant better improvement in student performance (Rouse, 2000).

Health care needs highly competent nurses. At the same time, the healthcare economy is strained. This is why time is a vital issue and cost‐benefit analyses are important. It is necessary to get as much knowledge as possible at a lower cost. In this study, the classroom‐taught participants spent more than twice as much time on the course as the e‐learning group did and not a single participant in the e‐learning group used as much time as scheduled in the conventional teaching group. This finding is in line with that of a study of nursing students in a nephrology course (Segal et al., 2013) and may imply that in e‐learning; it is possible to adapt one's learning pace and duration to one's former knowledge and thereby spend less time on learning. In classroom teaching, participants have to adapt to the lecturer's progress and other students' learning needs. By contrast, e‐learning makes it possible to adapt the amount of study time to one's former experience and knowledge, learning capacity and ability to concentrate over time. In classroom instruction, teachers may suspect that course attendees have varying baseline knowledge and cannot risk skipping the basics.

The participants in the e‐learning group were significantly less positive towards the learning method than the traditional learning group. When asked to rate their course on a 0–4 scale, the traditional learning group participants were significantly more positive than the e‐learning group participants: their respective average scores were 2.8 and 3.5 (*p* = 0.023).

The social aspects of traditional learning include the opportunity to discuss the curriculum and what was taught with an expert, and other course participants: this may be important for student satisfaction and well‐being (Liu et al., 2016). The less positive perception of e‐learning may therefore be due to the demand to work independently, making e‐learning “isolated learning.” This disadvantage was demonstrated in a report on a web‐based course on cardiac rhythm interpretation, where nurse students who were allowed to discuss ambiguous cases with each other were found to be more satisfied than those working independently and learning alone (Frith & Kee, 2003). Furthermore, participants' evaluations of courses may be affected by teacher showmanship as well as by student satisfaction with learning outcome (Kozub, 2008), as shown in a study where face‐to‐face taught students reported higher course satisfaction, but the online learning group knowledge scores improved more (Williams & Ceci, 1997). This may be a general bias regarding evaluations of learning methods (Kozub, 2008).

In this study, learning outcome was measured by a multiple‐choice test. Such knowledge is the basis of understanding state‐of‐the‐art CHD care and neonate needs, but patient outcome is also closely related to nurse competence and skills. As emphasized by the Bologna framework for higher education, learning outcomes are not only theoretical knowledge, but also practical skills and reflective competence (Cedefop, 2015; Norwegian Agency for Quality Assurance in Education, 2011). This study reports curriculum knowledge only and only in the short‐term context. It did not measure long‐term knowledge improvement nor to which degree theoretical knowledge was transferred to practical CHD nursing.

The fact that e‐learning was the less time‐consuming learning method and that traditional learning was the preferred learning method may imply that blended learning, mixing traditional learning and e‐learning may be the most effective learning method, as suggested by several researchers, for example Horiuchi, Yaju, Koyo, Sakyo, and Nakayama (2009), Means, Toyama, Murphy, and Baki (2013) and Liu et al. (2016). This may address both the need for time effectiveness and the need for interaction with a teacher or an expert.

In Norway, medical examination and surgical treatment of infants with severe CHD are clustered at one hospital, but the infants are followed up at 11 different hospitals around the country. As Norway is long and sparsely populated, decentralized classroom instruction means a shortage of CHD lecturers in NICUs. The CHD e‐learning modules used in this study are now available as courseware to nurses in hospitals nationwide. Because of various availability of CHD experts throughout the country, hospitals may choose different strategies to increase knowledge. Some use the developed e‐learning courses only and other include a combination of the e‐learning courses and lectures or as flipped classroom learning. In addition, the availability of the e‐learning courses may give healthcare professionals an opportunity to refresh their knowledge at any given time. Similar approaches to teach CHD with flexible methods may be useful in other communities or settings where an e‐learning program is tested and found useful.

### 5.1 Limitations

The major limitation of this study is its small sample size. The insignificance of the learning outcome difference between groups may reflect a lack of statistical power. On “reversing” the sample size calculation by considering the subgroup sizes as given and treating the *z*_beta_ as the equation's unknown parameter, our statistical power to detect a difference (*p* ≤ 0.05) of four points (13.6 vs. 9.6, as in our data set) was 71%. Before concluding which of our learning formats was better, a larger study must be undertaken. Other limitations are the lack of follow‐up data on long‐term outcomes and the lack of testing the participants' level of clinical skills, competencies and critical thinking based on their new knowledge.

## 6 CONCLUSION

The results of this study did not prove any of the learning methods—classroom teaching and e‐learning—more effective than the other in increasing knowledge scores on haemodynamics and how to provide nursing care for infants with CHD.

Both learning groups scored significantly higher 1 week after training. Controlled for course participants' number of year of experiences in CHD nursing and for baseline knowledge score, the improvement in the E‐learning group did not differ significantly from improvement in the traditional learning group. Participants reported experiencing traditional classroom teaching as more positive, but E‐learning may be more time effective. More research is necessary to evaluate which method that provides enduring knowledge gain and improves clinical work in line with the triple aim of the EU Bologna framework for professional education: not only knowledge, but reflective capacity and practical skills.

## CONFLICT OF INTEREST

The authors declare no conflict of interest.

## AUTHOR CONTRIBUTIONS

Elin Hjorth‐Johansen: conception, design, data collection, analyses and drafting the manuscript. Dag Hofoss: analyses and drafting the manuscript. Nina Margrethe Kynø: conception, design, data collection, analyses and drafting the manuscript.

## ETHICAL APPROVAL

In this study participants signed informed consent forms and the study was approved by the hospital’s privacy protection supervisor.
